# Supplementary material for: Modeling glioblastoma heterogeneity as a dynamic network of cell states
Source: Mol Syst Biol. 2021 Sep 16;17(9):e10105. doi: 10.15252/msb.202010105 (PMC8444284; doi:10.15252/msb.202010105)
Supplement: Supplementary file 6 — Source Data for Figure 5 [file MSB-17-e10105-s004.zip › Figure5A_sourcedata/GSEA_3071/hallmarks_stateII.GseaPreranked.1623742536692/HALLMARK_SPERMATOGENESIS.html]

Details for gene set HALLMARK\_SPERMATOGENESIS[GSEA]

|  || Dataset | state23071 |
| Phenotype | NoPhenotypeAvailable |
| Upregulated in class | na\_pos |
| GeneSet | HALLMARK\_SPERMATOGENESIS |
| Enrichment Score (ES) | 0.44064122 |
| Normalized Enrichment Score (NES) | 1.9230756 |
| Nominal p-value | 0.002247191 |
| FDR q-value | 0.006801074 |
| FWER p-Value | 0.039 |
Table: GSEA Results Summary

  

Fig 1: Enrichment plot: HALLMARK\_SPERMATOGENESIS      
 Profile of the Running ES Score & Positions of GeneSet Members on the Rank Ordered List

  

| PROBE | GENE SYMBOL | GENE\_TITLE | RANK IN GENE LIST | RANK METRIC SCORE | RUNNING ES | CORE ENRICHMENT || 1 | CDK1 |  |  | 14 | 1.540 | 0.0825 | Yes |
| 2 | KIF2C |  |  | 73 | 1.132 | 0.1315 | Yes |
| 3 | NF2 |  |  | 103 | 1.036 | 0.1822 | Yes |
| 4 | NCAPH |  |  | 116 | 1.013 | 0.2358 | Yes |
| 5 | EZH2 |  |  | 233 | 0.839 | 0.2542 | Yes |
| 6 | DBF4 |  |  | 240 | 0.832 | 0.2992 | Yes |
| 7 | AURKA |  |  | 360 | 0.705 | 0.3094 | Yes |
| 8 | TTK |  |  | 365 | 0.701 | 0.3475 | Yes |
| 9 | PSMG1 |  |  | 372 | 0.696 | 0.3850 | Yes |
| 10 | RFC4 |  |  | 385 | 0.686 | 0.4203 | Yes |
| 11 | PARP2 |  |  | 451 | 0.650 | 0.4406 | Yes |
| 12 | NEK2 |  |  | 1100 | 0.417 | 0.3048 | No |
| 13 | BUB1 |  |  | 1145 | 0.408 | 0.3167 | No |
| 14 | TOPBP1 |  |  | 1355 | 0.366 | 0.2859 | No |
| 15 | HSPA2 |  |  | 1688 | 0.312 | 0.2217 | No |
| 16 | MAST2 |  |  | 1699 | 0.311 | 0.2366 | No |
| 17 | SLC12A2 |  |  | 1712 | 0.309 | 0.2509 | No |
| 18 | ARL4A |  |  | 2167 | 0.255 | 0.1537 | No |
| 19 | HSPA4L |  |  | 2203 | 0.252 | 0.1591 | No |
| 20 | AGFG1 |  |  | 2711 | -0.318 | 0.0524 | No |
| 21 | BRAF |  |  | 2825 | -0.341 | 0.0436 | No |
| 22 | JAM3 |  |  | 3016 | -0.378 | 0.0180 | No |
| 23 | DCC |  |  | 3155 | -0.415 | 0.0073 | No |
| 24 | SPATA6 |  |  | 3606 | -0.608 | -0.0693 | No |
| 25 | PEBP1 |  |  | 3694 | -0.679 | -0.0528 | No |
| 26 | GAD1 |  |  | 3704 | -0.690 | -0.0164 | No |
| 27 | PIAS2 |  |  | 3848 | -0.840 | -0.0047 | No |
| 28 | PCSK1N |  |  | 3987 | -1.179 | 0.0273 | No |
Table: GSEA details [plain text format]

  

Fig 2: HALLMARK\_SPERMATOGENESIS: Random ES distribution      
 Gene set null distribution of ES for **HALLMARK\_SPERMATOGENESIS**
